# At what institutions did Nobel laureates do their prize-winning work? An analysis of biographical information on Nobel laureates from 1994 to 2014

**DOI:** 10.1007/s11192-016-2059-2

**Published:** 2016-07-25

**Authors:** Elisabeth Maria Schlagberger, Lutz Bornmann, Johann Bauer

**Affiliations:** 1 Max Planck Institute of Biochemistry, Am Klopferspitz 18, 82152 Martinsried, Germany; 2 Division for Science and Innovation Studies, Administrative Headquarters of the Max Planck Society, Hofgartenstr. 8, 80539 Munich, Germany

**Keywords:** Nobel Prize, Decisive work, Researcher mobility, Landmark papers, Affiliations

## Abstract

In this study we examined the institutions (and countries) the Nobel laureates of the three disciplines chemistry, physics and physiology/medicine were affiliated with (from 1994 to 2014) when they did the decisive research work. To be able to frame the results at that time point, we also looked at when the Nobel laureates obtained their Ph.D./M.D. and when they were awarded the Nobel Prize. We examined all 155 Nobel laureates of the last 21 years in physics, chemistry, and physiology/medicine. Results showed that the USA dominated as a country. Statistical analysis also revealed that only three institutions can boast a larger number of Nobelists at all three time points examined: UC Berkeley, Columbia University and the Massachusetts Institute of Technology (MIT). Researcher mobility analysis made clear that most of the Nobel laureates were mobile; either after having obtained their Ph.D./M.D. or after writing significant papers that were decisive for the Nobel Prize. Therefore, we distinguished different ways of mobility between countries and between institutions. In most cases, the researchers changed institutes/universities within one and the same country (in first position: the USA, followed, by far, by the United Kingdom, Japan and Germany).

## Introduction

The Nobel Prize is the most prestigious and renowned research prize for outstanding contributions in physics, physiology/medicine, literature, and peace, and it attracts widespread attention not only within but also outside the world of academia and science (www.nobelprize.org). Since research prizes in general (and the Nobel Prize specifically) can be used as indicators of research achievements, and as information on research prizes is usually well accessible, numerous scientometric studies investigating Nobel laureates have been conducted. For example, Zhou et al. (2014) examined 362 landmark papers written by Nobel laureates in physics from 1901 to 2012 using bibliometric methods (Journal Impact Factor, citations of landmark papers, country where the journal is published). In two recent studies, Chan et al. (2015a) and (b) looked at alterations of co-authors on the laureates’ publications, and Wagner et al. (2015) compared Nobel laureates with a matched group of scientists to examine productivity, impact, and research networks. In further studies, publications by Nobel laureates have been used, for example, to validate (newly suggested) bibliometric indicators (Antonakis and Lalive 2008; Aziz and Rozing 2013; Rodríguez-Navarro 2011a, b), to test the quality of Google Scholar as a source for citation data (Harzing 2013; Patel et al. 2013), to predict the awarding of Nobel Prizes (Ashton and Oppenheim 1978), to study the uncitedness of publications by reputable scientists (Egghe et al. 2011; Heneberg 2013), to determine the effect of the Nobel Prize on the citation impact of publications by a Nobel laureate (Frandsen and Nicolaisen 2013; Gingras and Wallace 2010; Mazloumian et al. 2011), and to investigate the relationship between the number of highly-cited papers and the awarding of the Nobel Prize (Chuang and Ho 2014; Laband and Majumdar 2012).

Previous studies not only examined Nobel Prizes and Nobel laureates using bibliometric methods, but also analyzed the event itself and the person as such from a sociology-of-science perspective. Becattini et al. (2014) studied the time delay between when a scientist makes a prize-winning discovery and is recognized for it with the Nobel Prize. They found that from the very beginning of the Nobel Prize awards, this lag time has continuously increased and Nobel Prize winners have become proportionally older and older at the time of their awards. On average, the lag time is almost twice as long in chemistry (9 years) and physiology/medicine (11 years) than in physics (5 years) (Chan and Torgler 2013). Before being awarded the Nobel Prize, most Nobel Prize winners received a striking number of other awards (Chan et al. 2014a, b) or were invited more frequently than other scientists to join scientific societies (Chan and Torgler 2012; Chan et al. 2016). In a series of studies, Campanario (1993, 1996, 2009) examined resistance in the Nobel laureates’ scientific communities to recognizing the later honored work (for example, a journal rejecting the paper on the later prize-winning work). Campanario’s studies clearly pointed out that rejections and resistance in the scientific community actually occurred. Stephan and Levin (1993), Jones and Weinberg (2011) looked at the age of Nobel Prize winners and examined the relationship between age and scientific productivity and creativity.

With this paper, we take up from one of the most important empirical studies in this area entitled “Scientific elite. Nobel laureates in the United States” by Zuckerman (1977). The author tracked all Nobel laureates in the USA awarded the prizes from 1901 to 1972. Zuckerman (1977) focused on the question, which social factors and social conditions “make” Nobel laureates. The author investigated their social development, educational background, collaboration with other authors, and the specialty of the prize-winning research. For this purpose, Zuckerman (1977) interpreted the data also on the basis of interviews with laureates (e.g. in order to explain the mobility of laureates who “moved” or “stayed”).

Zuckerman (1977) examined the prize-winning research, looking at US research institutions where the prize-winning work was done (see Zuckerman 1977, p. 170, table 6–3). Such research institutions are of special interest generally, because it is considered that they provide very good research conditions. A similar research approach was used by Ye et al. (2013), who analyzed the awards of 66 Nobel laureates in physiology/medicine during the period from 1983 to 2012. They reported one to at most four landmark papers describing the work decisive for Nobel Prizes. Furthermore, they listed the journal of the most cited work (see Ye et al. 2013, p. 536, Table 2). Knowing the institution with which the researcher is affiliated at the time of doing the decisive work is important, because many Nobel laureates were awarded the Nobel Prize many years after they did the prize-winning work, and because researchers tend to be mobile—that is, they often change research institutions.

Besides Zuckerman (1977), we take up from another important empirical study in the area of Nobel Prize analysis: Hillebrand (2002) undertook a biographical analysis of the Nobel laureates in physics from 1901 to 2000. In the analysis, he considered information about the Nobel laureates, e.g. age, teamwork or migration within countries. He focused on the laureates’ curriculum vitae, e.g. their social responsibilities. The author concluded that “success is made more likely by an early interest in science, a good education, hard work, mobility (on occasion), as well as a generous portion of luck” (Hillebrand 2002, p. 93).

In this study we examined the institutions the Nobel laureates of the three disciplines chemistry, physics and physiology/medicine were affiliated with (from 1994 to 2014) when they did the decisive research work. To be able to compare the results, we also identified the Nobel laureates’ institutional affiliations at the time point of their obtaining a Ph.D./M.D. and at the time point of their being awarded the Nobel Prize. In addition, we examined the Nobel laureates’ mobility across the three time points.

## Methods

### Sources

The names of all Nobel laureates and their institutional affiliations on the day of the Prize announcement were found on the Nobel Prize website, www.nobelprize.org. The website also provides a broad summary statement naming the research achievement or discovery for which the prize was awarded as well as information on whether the prize was awarded to one person or shared by two or three persons maximum. This summary statement was the most important and the only (official) indication of the honored research work/papers of the Nobel laureates.

In the three prize categories (physiology/medicine, chemistry, physics), 155 scientists were awarded the Nobel Prize in the 21 years from 1994 to 2014. Compared to the prize categories physiology/medicine and chemistry, most prizes (55) were awarded in physics. There were 50 Nobel laureates each in physiology/medicine and chemistry.

None of the Nobel laureates examined here was awarded a second Nobel Prize, which has occurred only four times in the past. But we found differences in the categories with regard to whether the Nobel Prize was awarded to one person or shared by two or three persons: whereas in the period from 1994 to 2014 the Nobel Prize for physics was shared by two or three persons every year (without exception), the Nobel Prize for chemistry was awarded to one person (unshared) five times (Georg A. Olah 1994, Ahmed H. Zewail 1999, Roger D. Kornberg 2006, Gerhard Ertl 2007, and Dan Shechtman 2011). In physiology/medicine, the prize was awarded to one person (unshared) three times (Stanley Prusiner 1997, Günter Blobel 1999, and Robert G. Edwards 2010).

In most cases, information on the date and place where the Nobel laureates obtained their Ph.D./M.D. was available in the *Encyclopædia Britannica* (see www.britannica.com). But the *Britannica* was mainly important as a reference work for information on the course of the Nobel laureates’ careers. The information summarized in *Encyclopædia Britannica* made it possible to narrow down the time frame in which the prize-winning work was done or to find out what the Nobel laureates’ major research achievement was. The *Encyclopædia Britannica* was the most important source of information next to the Nobel Prize website. However, as there are sometimes gaps in accounts of the Nobel laureates’ careers in the *Encyclopædia Britannica*, it made sense to also consult current university and institute web pages, which in most cases provide information on their Nobel laureates. In this way, information was obtained on the workplaces of all 155 Nobel laureates examined.

### Determining the prize-winning work

There is no one decisive publication as such, which could clearly indicate the affiliation, where the laureate did his decisive work. The Nobel Committees did not name any relevant publications as official justification. However, the summary statement issued by the Committee (www.nobelprize.org), naming the research achievement or discovery for which a Nobel Prize was awarded, narrows down the topic of the prize-winning work.

Utilizing the Nobel Committee’s reason for which the prize was awarded and the information on a laureate’s career taken from the *Encyclopædia Britannica*, we identified the paper(s) in which the researcher described the prize-winning work. We searched for landmark papers of the laureates in literature databases such as Web of Science (WoS, Thomson Reuters) or Scopus (Elsevier), that—among other things—capture citation counts and the addresses of authors of publications (Scopus since 1996). To determine lacking publications about the prize-winning work, we searched for books via Google Scholar and used further databases (e.g. ProQuest or Wiley Online Library). For example, K. Tanaka’s (Nobel Prize 2002 in chemistry) prize-winning paper (Tanaka et al. 1988) was found neither in WoS nor in Scopus, but in Google Scholar.

In any case, we took the author’s institutional affiliation named in the paper as the institution where the researcher wrote the prize-winning publication. Almost always, not just one paper, but rather several papers on the same topic (up to five) came into question. Based on the one or several relevant publications, we then determined the institutions where the Nobel laureates were working when they gained the important research results.

A difficulty encountered in this search was that, in some cases, Nobel laureates published several papers on the relevant topic within the same year. In that case, we used further analysis methods. After searching WoS and Scopus for all publications by the correct author (established clearly by using initials, biography, etc.), we examined the content of the most highly cited papers more closely by reading the abstract and, when necessary, checking the full text as well.

## Results

### The characterization of the prize-winning publication(s)

As to the document type of publication, we found that in most cases (about 95 %), the prize-winning work was published in the form of an ‘article,’ which is the most relevant document type in the natural sciences. Occasionally, we found signed letters as a document type for the prize-winning work of F. Englert, (Nobel Prize 2013 in physics) in Englert and Brout (1964), for the laureate’s work of H. Kroemer (Nobel Prize in physics 2000) in Kroemer (1963) and for the prize-winning work of P. Mansfield (Nobel Prize 2003 in physiology/medicine) in Mansfield (1977). In addition, we found two book chapters by J. O’Kneefe (Nobel Prize 2014 in physiology/medicine) in O’Keefe and Nadel (1978) and by G. Blobel (Nobel Prize 1999 in physiology/medicine) in Blobel and Sabatini (1971). Two further papers were published as a meeting abstract—R. F. Furchgott (Nobel Prize 1998 in physiology/medicine) in Furchgott et al. (1987)—and as note—P. A. Grünberg (Nobel Prize 2007 in physics) in Binasch et al. (1989). In physics, at least one researcher, J. Kilby, was awarded the Nobel Prize, who applied in 1959 for a patent using his work on the invention of the world’s first integrated circuit (IC) chip (Kilby 1959, Patentnumber:US3072832). He worked at Texas Instruments Inc. (Bell liscensee) in Dallas (TX) as an employee from 1958 to 1970.

The results show that in addition to publications in renowned scientific journals like *Nature*, *Science*, *Cell* and *Physical Review Letters*, less well-known journals also helped the way to the Nobel Prize. For example, we found a non-English publication (in French) of Y. Chauvin (Nobel Prize 2005 in chemistry) in Herisson and Chauvin (1971).

Regarding the content of the prize-winning work of all 155 Nobel laureates, we defined three categories: (1) development of methods, e.g. useful in medical diagnosis or chemical synthesis, (2) discoveries of natural mechanism and phenomena, and (3) making mature products. In our analysis, medicine leads with 18 discoveries of natural mechanism (85.7 %), followed by physics with 15 (71.4 %) and chemistry with 10 (47.6 %). In contrast, chemistry ranks first with nine methodical prize-winning works (42.9 %), followed by physics with seven (33.4 %) and physiology/medicine with three (14.3 %). The definition of a mature product is an industrial product development, like the invention of LED-lights (Nobel Prize in physics 2014, Nakamura, Amano and Akasaki) and “for basic work on information and communication technology” (Nobel Prize in physics 2000, Kroemer, Zhores, Kilby). Mature products were seldom described in decisive publications; only chemistry and physics hold two prize-winning works (both 9.5 %), physiology/medicine has none.

A peculiarity in the awarding of the Nobel Prize during the examined time period in both physics and chemistry was of interest: Six scientists with a university degree but without a Ph.D. or M.D. were awarded a Nobel Prize: Koichi Tanaka (Nobel Prize in chemistry 2002), Yves Chauvin (Nobel Prize in chemistry 2005), Barry Marshall and Robin Warren (Nobel Prizes in physiology/medicine 2005), Jack Kilby (Nobel Prize in physics 2000), and Shuji Nakamura (Nobel Prize in physics 2014).

### Institutions with which the Nobel laureates were affiliated

Table 1 lists the institutions at which Nobel laureates of the last 21 years obtained their Ph.D./M.D., at which they made their prize-winning discovery, and with which they were affiliated at the time of the Nobel Prize award. In order to see whether the institutions listed in the table are also top-rated institutions in university rankings, we included the ranking positions provided by Claassen (2015, Fig. 2, p. 800). Claassen (2015) presents a meta-ranking of universities which is based on major university rankings. The comparison shows that—with the exception of the Nagoya University—ranking positions below 30 are more frequently among the institutions where the Ph.D./M.D. was obtained than among the institutions where the prize-winning work was done or the Nobel Prize awarded.

**Table 1:** Number of Nobel laureates affiliated with the listed institutions when they obtained their Ph.D./M.D., did the prize-winning work, and were awarded the Nobel Prize

Career stage	Institution	Number of scientists	Ranking results (Claassen 2015)
Ph.D./M.D. obtained	Harvard University, USA	14	1
Gini = 0.24**	University of California, Berkeley, USA	8	5
	MIT, Cambridge, MA, USA	6	2
	University of Cambridge, U.K	5	4
	Nagoya University, Nagoya, Japan	5	152
	University of Oxford, Oxford, U.K	5	6
	Yale University, New Haven, USA	5	11
	Columbia University, New York, USA	4	8
	Cornell University, Ithaka, USA	4	13
	Stanford University, Stanford, USA	4	3
	New York University, New York, USA	3	28
	John Hopkins, Baltimore, USA	3	15
Did the prize-winning work	Cambridge University, U.K	8	4
Gini = 0.17**	University of California, Berkeley, USA	6	5
	AT&T, Bell Labs, Murray Hill, USA	6	Not included
	MIT, Cambridge, MA, USA	3	2
	Rockefeller University, New York, USA	4	42
	Harvard University, USA	4	1
	Univ. Pennsylvania, Philadelphia, USA	3	14
	Technion—Israel, Haifa, Israel	3	148
	Cornell University, Ithaka, USA	3	13
	Rice University Houston	3	91
	Kyoto University, Kyoto, Japan	3	30
	University of Colorado, Boulder, USA	3	44
	Yale University, USA	3	11
	University of Columbia, New York, USA	3	8
	Nagoya University, Nagoya, Japan	3	152
	California Institute of Technology (Caltech), Pasadena, USA	3	Not included
Nobel Prize awarded	Stanford University, Stanford, USA	10	3
Gini = 0.19**	MIT, Cambridge, MA, USA	6	2
	University of California, Santa Barbara, USA	5	37
	University of California, Berkeley, USA	4	5
	Max-Planck-Society, Germany	4	Not included
	Columbia University, New York, USA	4	8
	Rockefeller University, New York, USA	4	42
	California Institute of Technology (Caltech), Pasadena, USA	4	Not included
	University of Colorado, Boulder, USA	3	44
	Technion—Israel, Haifa, Israel	3	148
	John Hopkins, Baltimore, USA	3	15
	National Inst. of Standards and Technology (NIST), Gaithersburg/Boulder USA	3	Not included

The table lists only institutions with which at least three persons were affiliated. Complete lists of Nobel laureates are shown in the Appendix Tables 5, 6 and 7

** A Gini coefficient of zero expresses equality among the values in a frequency distribution; a Gini coefficient of one maximum inequality

We also calculated Gini coefficients as a measure of statistical dispersion for the number of scientists in Table 1. These coefficients indicate that the scientists are more equally distributed among the institutions where the prize-winning work was done (Gini = 0.17) and the Nobel Prize awarded (Gini = 0.19) than among the institutions where the Ph.D./M.D. was obtained (Gini = 0.24). Obviously, certain institutions (like Harvard University) are not only able to recruit promising Ph.D./M.D. candidates, but offer also fruitful environments for starting a successful career in science.

In detail, Table 1 shows that most of the Nobel laureates obtained their Ph.D./M.D. in the USA at Harvard University (n = 14), the University of California, Berkeley (UC Berkeley) (n = 8), and Massachusetts Institute of Technology (MIT) (n = 6). Harvard University stood out from the others by far; with almost twice as many future Nobel laureates as UC Berkeley, it fulfilled the criterion as the most important university for future Nobel laureates.

Regarding affiliations while doing the relevant prize-winning work/paper, no single institution stood out with a very high number of persons: leading the list of institutions here were Cambridge University (U.K.) (n = 8), followed by UC Berkeley and Bell Laboratories, or Bell Labs (formerly AT&T Bell Laboratories and Bell Telephone Laboratories), with six persons each. Bell Labs, which took on the research functions for the American Telephone and Telegraph (AT&T) company—a North American telecommunications company—is one of the few companies that can boast Nobel laureates (see http://ethw.org/Bell_Labs).

The research institutions with which a Nobel laureate was most frequently affiliated at the time of the Nobel Prize award were Stanford University (n = 10) and MIT (n = 6) (see Table 1). Looking at the research institutions across the different stages of the scientists’ careers, there were only three institutions that fulfilled the criterion at all three time points (Ph.D./M.D., prize-winning work/paper, Nobel Prize): UC Berkeley, Columbia University and MIT. A number of institutions were on the list for two of the three time points (such as Harvard University, Cambridge University U.K., Yale University and Technion-Israel).

The results make clear that the Nobel laureates did their prize-winning work/paper mainly at institutions in the USA. Still, the results differ greatly regarding the institution at which the prize-winning work was done: in Zuckerman’s (1977) study, the two most frequent institutions where the prize-winning work was done were Harvard University (n = 13) and Columbia University (n = 9), but in this study they were Cambridge University U.K. (n = 8), University of California, Berkeley (n = 6) and AT&T Bell Labs (n = 6) (see Zuckerman 1977, p. 171, table 6-3). In this study, only four Nobel laureates did their prize-winning work at Harvard University. The differences in the results of both studies are probably due to the fact that the time periods investigated in the studies were not of the same length and also that the historical and social contexts were different: Zuckerman (1977) looked at the period from 1901 to 1972, whereas this study examined the period from 1994 up to 2014. Similar to the results of this study are the results by Charlton (2007), who examined revolutionary biomedical science between 1992 and 2006 using Nobel Prizes, Lasker awards (clinical medicine) and Gairdner awards. However, Charlton’s (2007) study only looked at the time point of the Nobel Prize award and the field of biomedicine. In first place with the greatest number of Nobel laureates, Charlton (2007) found with n = 6 for MIT a similar number as this study, but also the University of Washington at Seattle (also with n = 6), which is not included in the list of institutions in this study (see Table 1).

In addition to examining the institutions with which the Nobel laureates were affiliated, we also looked at countries for the three time points of Ph.D./M.D., work/paper, and the Nobel Prize award. Table 2 shows the distribution of the 155 Nobel laureates in different countries at the three time points. As in Table 1, we listed only those countries in which we found at least three Nobel laureates. The table shows clearly that the USA was the country having apparently the best conditions for promoting a Nobel Prize winner. A similar result was reported by two other studies, which examined Nobel laureates in biomedicine (Charlton 2007) and economics (van Dalen 1999). Chan and Torgler (2015), who looked at Nobel laureates in physics, chemistry, and physiology/medicine between 1900 and 2000, found that “researchers educated in Great Britain and the US tend to attract more awards than other Nobelists” (p. 847).

**Table 2:** Number of future Nobel laureates affiliated with the listed countries when they obtained their Ph.D./M.D., did the prize-winning work, and were awarded the Nobel Prize (the table lists only countries with which at least three persons were affiliated)

Career stage	Country	Number of scientists
Ph.D./M.D. obtained*	USA	79+$
	United Kingdom	19.5+
	Japan	12
	Germany	10.5$
	France	7
	Israel	5
	Russia	4
	Canada	3
	The Netherlands	3
Did the prize-winning work*	USA	90
	United Kingdom	17
	Japan	10
	France	7
	Germany	6.5++
	Australia	5
	Israel	4.5++
	Russia	3
Nobel Prize awarded*	USA	98
	United Kingdom	14.5
	Japan	10
	Germany	7
	France	7.5
	Israel	4
	Australia	3

+ MacDiarmid received two Ph.D.s, first at the University of Wisconsin-Madison, USA and later at Cambridge University, U.K

$ Günter Blobel received his M.D. in Germany and subsequently his Ph.D. in the USA

++ Ada Yonath performed her decisive work in Israel and Germany

* Several Nobel Prize winners indicated two addresses

Table 2 illustrates an interesting finding for Japan. Compared to other countries, Japan is very well placed, although previous bibliometric studies found that Japan does not perform well on field-normalized citation impact (Bornmann and Leydesdorff 2013). Even though the number of Nobel laureates in a country and citation impact is used as indicators (proxies) for measuring the quality of research, they appear to measure different aspects of quality.

### Nobel laureates’ mobility

As described above, the different institutions counted different numbers of Nobel laureates when they obtained their Ph.D./M.D., did the prize-winning work/paper, and received the Nobel Prize. This result points to considerable mobility on the part of the Nobel laureates.

Table 3 visualizes their institutional mobility, distinguishing five types of institutional mobility behavior:

**Table 3:** Nobel laureates’ changes of affiliations

Type of mobility	Chemistry	Medicine/physiology	Physics	Total	%	Cumulative %
1	5	3	6	14	10.4	10.4
2	19	17	22	58	43.0	53.4
3	15	21	12	48	35.5	88.9
4	3	5	7	15	11.1	100
5	0	0	0	0	0

1. The Nobel laureates were affiliated with one and the same institution across the three career stages (Ph.D./M.D., prize-winning work/paper, Nobel Prize award).
2. The Nobel laureates obtained a Ph.D./M.D. at one institution and then moved on to another institution, with which they were affiliated while doing their prize-winning work/paper, and received the Nobel Prize.
3. The Nobel laureates obtained a Ph.D./M.D. at one institution and then moved to another institution, where they did their prize-winning work/paper. They received the Nobel Prize while affiliated with a third institution.
4. The Nobel laureates obtained a Ph.D./M.D. and did their prize-winning work/paper at one institution. They received the Nobel Prize while affiliated with another institution.
5. The Nobel laureates obtained their Ph.D./M.D. at one institution and then moved to another institution, where they did their prize-winning work/paper. They then returned to the first institution, with which they were affiliated when they received the Nobel Prize.

Table 3 shows the Nobel laureates’ changes of affiliations. The counts show clearly that only 10.4 % of the Nobel laureates remain at the same place during their entire career. The rest were mobile either after obtaining their Ph.D./M.D. namely 78.5 % or after doing their prize-winning work namely 89.6 %. These percentages suggest that successful careers are related to mobility.

We also looked at mobility on a country basis, since the Nobel laureates changed institutions not only within a country but also across countries. Again, we found five different types of mobility:

1. The Nobel laureates were in one and the same country across their three career stages (Ph.D./M.D., prize-winning work/paper, Nobel Prize award).
2. The Nobel laureates obtained a Ph.D./M.D. in one country and then moved to another country, where they did their prize-winning work/paper and received the Nobel Prize.
3. The Nobel laureates obtained their Ph.D./M.D. in one country and then moved to another country, where they did their prize-winning work/paper. They received the Nobel Prize while they were working in a third country.
4. The Nobel laureates obtained a Ph.D./M.D. and did their prize-winning work/paper in one country. They received the Nobel Prize while they were working in another country.
5. The Nobel laureates obtained their Ph.D./M.D. in one country and then moved to another country, where they did their prize-winning work/paper. They then returned to the first country, where they received the Nobel Prize.

Table 4 shows the Nobel laureates’ mobility behavior across countries. The results show that a large part of the Nobel laureates, 77 % (see Table 4), were not mobile across countries and worked in only one country during all time points.

**Table 4:** Nobel laureates’ mobility behavior across countries

Type of mobility	Chemistry	Medicine/physiology	Physics	Total	%	Cumulative %
1	36	38	40	114	77	77
2	2	4	10	16	10.8	87.8
3	1	1	0	2	1.4	89.2
4	3	3	4	10	6.8	96
5	3	3	0	6	4	100

Comparing Tables 3 and 4 indicates that Nobel laureates most frequently changed institutions within a given country. This fits the observation by Hillebrand (2002): “Since 1950, almost all laureates have remained in the country in which they made their discovery” (p. 89). This sedentariness is not only a characteristic of laureates, but also of common researchers. According to the results of Elsevier and Science Europe (2013) “the most common mobility class in both Europe and the US is sedentary; that is, researchers with published outputs reflecting only affiliation(s) within a single European country or within a single US state during the period 1996–2011 inclusive” (p. 30).

## Discussion

In modern science, evaluation of research is an increasingly important topic (Dahler-Larsen 2011; Power 1999). Whereas in the past the interest was in evaluating research presented in manuscripts or research proposals, today, entire institutions, research clusters and countries are being examined using indicators. Bibliometric indicators are certainly the most important class of indicators used (Moed 2005). With the aid of the underlying publication and citation data, evaluations can be done at any aggregation level—that is, from the individual researcher to entire continents. Bibliometric indicators can measure only the impact of science on science itself. But since science policy is interested in impact measurement above and beyond science and in other parts of society, scientometrics research is working on issues to measure this broader impact (Bornmann 2012, 2013).

Research is conducted by persons. As research prizes are usually awarded to persons who have made outstanding scientific achievements, the prizes are also used as indicators of research performance (Rodríguez-Navarro 2011, 2015).

As opposed to bibliometrics, however, the criterion of a research prize has two major disadvantages that make it difficult to use research prizes as indicators:

1. Because citation rates vary widely across disciplines and the variation has little to do with scientific quality, citation scores are normalized for this difference across disciplines (Vinkler 2010). Only through normalizing the impact scores of research institutions (and other entities), conduct research and publishing in different disciplines can be compared to one another. Since it can be assumed that there are discipline-specific patterns also with research prizes (many prizes are awarded only in a specific field), a comparison of results on the number of research prizes per institutions having different disciplinary profiles is not possible. To our knowledge, no methods for producing normalized numbers of research prizes yet have been suggested (if they are possible at all).
2. The awarding of research prizes, especially Nobel Prizes, is a rather rare event. A number of conditions have to be met for a research prize to be awarded, and not all of these conditions have to do with research quality. It can therefore be assumed that with the Nobel Prize there have been many false negatives: a number of important scientists that actually deserved the prize for their research findings or discoveries did not win a Nobel Prize for the various reasons (that had scarcely to do with research quality). The results of this study demonstrate that the research prize as a rare event is based on a small numbers of cases—even when the data is evaluated at the level of countries. With only a small number of cases, there is always the risk that results will be unreliable.

For these reasons, the results of this study should be handled with caution if they are used in an evaluative context. They may better be used to draw the public’s attention to topics investigated by the laureates (Chan et al. 2014a, b).

In this study we looked at the institution and country where a Nobel laureate did the work and was later awarded a Nobel Prize. To better understand the results of this time point, we in addition examined the number of Nobel laureates at the time points of obtaining their Ph.D./M.D. and when receiving the Nobel Prize. The results of the country analysis revealed that the USA dominates the country ranking. The institutional analysis shows that three institutions have a large number of Nobel laureates at all three time points: UC Berkeley, Columbia University and the Massachusetts Institute of Technology (MIT). The mobility analysis made clear that most of the Nobel laureates were mobile either after obtaining their Ph.D./M.D. or after doing the prize-winning work/paper. In most cases, the researchers moved from one institution to another within the same country (in the USA).

Explaining their individual motivations for moving or staying (using methods of qualitative research) could be a topic for future studies.

Despite the low numbers of events (n = 155 laureates) the results of this study in part resemble the findings gained by other studies. Analyzing citation impact, it was shown by Bornmann and Leydesdorff (2013) that countries such as USA, U.K., and Germany, having a high population number and a well working economy, lead in science. Bornmann and Bauer (2015) evaluated the list of 3216 researchers, who met the criteria of being highly cited researchers based on papers published between 2002 and 2012. They determined the number of highly cited researchers per institution and came to similar institutional rankings as shown in this study. A major difference to the current study is the fact that the Chinese Academy of Sciences ranks in the top ten of highly cited researchers in contrast to none Chinese Nobel laureate in one of the three disciplines before 2015 (Y. Tu received the Nobel Prize in physiology/medicine in 2015). We expect to see more Nobel laureates from China in the near future.

In this study, we examined a time point that was hardly previously analyzed: the career stage during which the researcher did the prize-winning work/paper. As laureates usually receive the Nobel Prize many years after this time point, the usual perspective in scientometrics, which attributes research achievements to institutions and countries at the award of the Nobel Prize, should be complemented by the perspective, which attributes achievements to the location where the researcher did the prize-winning work/paper.

## Appendix

See Tables 5, 6 and 7.

**Table 5:** Detailed information on Nobel laureates in chemistry

Year of award	Name	Justification	Ph.D./M.D. obtained	Did the prize-winning work(s)	Nobel Prize awarded
1994	Olah, George A.	Olah “for his contribution to carbocation chemistry”	Technical University, Budapest, Ungarn	Dow Chemical Company (Sania, Ontario/Canada)	University of Southern California (USC), Los Angelas, USA
1995	Crutzen, Paul J.	Crutzen, Molina, Rowland “for their work in atmospheric chemistry, particularly concerning the formation and decomposition of ozone”	University of Stockholm, Sweden (Ph.D. and Dr. Science)	University of Stockholm, Sweden	Max-Planck-Institute f. Chemistry, Mainz, Germany
	Molina, Mario J.	Molina, Crutzen, Rowland “for their work in atmospheric chemistry, particularly concerning the formation and decomposition of ozone”	University of California, Berkeley, USA	University of California Irvine (UCI), USA	Massachusetts Institute of Technology (MIT), MA, USA
	Rowland, Frank S.	Rowland, Crutzen, Molina “for their work in atmospheric chemistry, particularly concerning the formation and decomposition of ozone”	University of Chicago, USA	University of California Irvine (UCI), USA	University of California Irvine (UCI), USA
1996	Curl (Jr.), Robert F.	Curl, Kroto, Smalley “for their discovery of fullerenes”	University of California, Berkeley, USA	Rice University, Houston, USA	Rice University, Houston, USA
	Kroto, Harold W.	Kroto, Curl, Smalley “for their discovery of fullerenes”	University of Sheffield, U.K	Rice University, Houston, USA	University of Sussex, Brighton, U.K
	Smalley, Richard E.	Smalley, Curl, Kroto “for their discovery of fullerenes”	Princeton University, New Jersey, USA	Rice University, Houston, USA	Rice University, Houston, USA
1997	Boyer, Paul D.	Boyer, Walker “for their elucidation of the enzymatic mech-anism underlying the synthesis of adenosine triphosphate (ATP)”	University of Wisconsin-Madison, Madison, USA	University California (UCLA), Los Angeles, USA	University California (UCLA), Los Angeles, USA
	Walker, John E.	Walker, Boyer “for their elucidation of the enzymatic mechanism underlying the synthesis of adenosine triphosphate (ATP)”	Oxford University, Sir William Dunn School of Pathology, U.K	Medical Research Council (MRC), Cambridge, U.K	Medical Research Council (MRC), Laboratory of Molecular Biology, Cambridge, U.K
	Skou, Jens C.	Skou “for the first discovery of an ion-transporting enzyme, Na+ , K+ -ATPase”	Aarhus University, Denmark	Aarhus University, Denmark	Aarhus University, Denmark
1998	Kohn, Walter	Kohn “for his development of the density-functional theory”	Harvard University, Cambridge, MA, USA	University of California, San Diego, USA	University of California, Santa Barbara, USA
	Pople, John A.	Pople “for his development of computational methods in quantum chemistry”	Cambridge University, U.K	Carnegie–Mellon University, Pittsburgh, USA	Northwestern University, Evanston, USA
1999	Zewail Ahmed H.	Zewail “for his studies of the transition states of chemical reactions using femtosecond spectroscopy”	University of Pennsylvania, Philadelphia, USA	California Institute of Technology (Caltech), Pasadena, USA	California Institute of Technology (Caltech), Pasadena, USA
2000	Heeger, Alan J.	Heeger, MacDiarmid, Shirkawa “for the discovery and development of conductive polymers”	University of California, Berkeley, USA	University of Pennsylvania, Philadelphia, USA	University of California, Santa Barbara, USA
	MacDiarmid, Alan G.	MacDiarmid, Heeger, Shirakawa “for the discovery and development of conductive polymers”	University of Wisconsin, Madison, USA and Cambridge University, U.K	University of Pennsylvania, Philadelphia, USA	University of Pennsylvania, Philadelphia, USA
	Shirakawa, Hideki	Shirakawa, MacDirmid, Heeger “for the discovery and development of conductive polymers”	Tokyo Institute of Technology, Japan	University of Pennsylvania, Philadelphia, USA	University of Tsukuba, Tokyo, Japan
2001	Knowles, William S.	Knowles, Noyori “for their work on chirally catalysed hydrogenation reactions”	Columbia University, New York, USA	Monsanto, St. Louis, USA	Monsanto, St. Louis, USA (retired)
	Noyori, Ryoji	Noyori, Knowles “for their work on chirally catalysed hydrogenation reactions”	Kyōto University, Japan	Nagoya University, Japan	Nagoya University, Japan
	Sharpless, K. Barry	Sharpless “for his work on chirally catalysed oxidation reactions”	Stanford University, CA, USA	Massachusetts Institute of Technology (MIT), Cambridge, USA	The Scripps Research Inst. La Jolla (TSRI), CA, USA
2002	Fenn, John B.	Fenn, Tanaka “for their development of soft desorption ionisation methods for mass spectrometric analyses of biological macromolecules”	Yale University, New Haven, USA	Yale University, New Haven, USA	Virginia University, Richmond, USA
	Tanaka, Koichi	Tanaka, Koichi “for their development of soft desorption ionisation methods for mass spectrometric analyses of biological macromolecules”	no Ph. (Tōhoku University, Tōhoku, Japan; engineering degree)	Shimadzu Corporation Kyōto, Japan	Shimadzu Corporation Kyōto, Japan
	Wüthrich, Kurt	Wüthrich “for his development of nuclear magnetic resonance spectroscopy for determining the three-dimensional structure of biological macromolecules in solution”	Basel University, Swiss	Eidgenössische Tech. Hochschule (ETH), Zürich, Swiss	Eidgenössische Tech. Hochschule (ETH), Zürich, Swiss and The Scripps. Research Institute La Jolla (TSRI), California, USA
2003	Agre, Peter	Agre, McKinnon “for discoveries concerning channels in cell membranes”; Agre “for the discovery of water channels”	John Hopkins, School of Medicine, Baltimore, USA	John Hopkins, School of Medicine, Baltimore, USA	John Hopkins, School of Medicine, Baltimore, USA
	MacKinnon, Roderick	MacKinnon, Agre “for discoveries concerning channels in cell membranes”; McKinnon “for structural and mechanistic studies of ion channels”	Tufts University, Medford, USA	Rockefeller University, New York, USA	Rockefeller University, New York, USA
2004	Ciechanover, Aaron	Ciechanover, Hersko, Rose “for the discovery of ubiquitin-mediated protein degradation”	Hebrew University–Hadassah, Jerusalem, Israel M.D. and Ph.S. at Technion–Israel Institute of Tech-nology, Haifa, Israel	Technion—Israel Institute of Technology, Haifa, Israel	Technion—Israel Institute of Technology, Haifa, Israel
	Hershko, Avram	Hersko, Chiechanover, Rose “for the discovery of ubiquitin-mediated protein degradation”	Hebrew University–Hadassah, Jerusalem, Israel M.D. and Ph.D.	Technion—Israel Institute of Technology, Haifa, Israel	Technion—Israel Institute of Technology, Haifa, Israel
	Rose, Irwin	Rose, Hersko, Chiechanover “for the discovery of ubiquitin-mediated protein degradation”	University Chicago, USA	FOX Chase Cancer Center, Philadelphia, USA	University California Irvine (UCI), USA
2005	Chauvin, Yves	Chauvin, Grubbs, Schrock “for the development of the metathesis method in organic synthesis”	No Ph. D. (degree L`École Chemie Industrielle, Lyon, France)	Institut Français du Pétrole, Rueil-Malmaison, (near Paris), France	Institut Français du Pétrole, Rueil-Malmaison, (near Paris), France
	Grubbs, Robert H.	Grubbs, Schrock, Chauvin “for the development of the metathesis method in organic synthesis”	Columbia University, New York, USA	California Institute of Technology (Caltech), Pasadena, USA	California Institute of Technology (Caltech), Pasadena, USA
	Schrock, Richard R.	Schrock, Grubbs, Chauvin “for the development of the metathesis method in organic synthesis”	Harvard University, Cambridge, MA, USA	Massachusetts Institute of Technology (MIT), Cambridge, MA, USA	Massachusetts Institute of Technology (MIT), Cambridge, MA, USA
2006	Kornberg, Roger D.	Kornberg “for his studies of the molecular basis of eukaryotic transcription”	Stanford University, CA, USA	Stanford University, CA, USA	Stanford University, CA, USA
2007	Ertl, Gerhard	Ertl “for his studies of chemical processes on solid surfaces”	Technische Universität (TU), Munich, Germany	Ludwig-Maximilians-Universität Munich, Germany	Fritz-Haber-Institut, Max-Planck-Institute, Berlin, Germany
2008	Shimomura, Osamu	Shimomura, Chalfie, Tsien “for the discovery and development of the green fluorescent protein, GFP”	Nagoya University, Japan	Princeton University, NJ, USA	Boston University Medical School, Marine Biological Laboratory (MBL), Woods Hole, MA, USA
	Chalfie, Martin	Chalfie, Shimomura, Tsien “for the discovery and development of the green fluorescent protein, GFP”	Harvard University, Cambridge, MA, USA	Columbia University, New York, USA	Columbia University, New York, USA
	Tsien, Roger Yonchien	Tsien, Chalfie, Shimomura “for the discovery and development of the green fluorescent protein, GFP”	Cambridge University, U.K	Cambridge University, U.K	University California, San Diego, USA
2009	Ramakrishnan, Venkatraman (also Venki)	Ramakrishan, Steitz, Yonath “for studies of the structure and function of the ribosome”	Ohio University, Athens, OH, USA	University of Utah, Salt Lake City, USA	Laboratory of Molecular Biology (MRC), Cambridge, U.K
	Steitz, Thomas A.	Steitz, Ramakrishan, Yonath “for studies of the structure and function of the ribosome”	Harvard University, Cambridge, MA, USA	Yale University, New Haven, USA u. HHMI, New Haven, USA (both affiliations)	Yale University, New Haven, USA
	Yonath, Ada E.	Yonath, Ramakrishan, Steitz “for studies of the structure and function of the ribosome”	Weizman Institute (WIS), Rehovot, Israel	Max-Planck Research Unit for Ribosome Structure, Hamburg, Germany and Weizmann Institute, Rehovot, Israel	Weizmann Institute of Science, Rehovot, Israel
2010	Heck, Richard Fred	Heck, Negishi, Suzuki “for palladium-catalyzed cross couplings in organic synthesis”	University California (UCLA), Los Angeles, USA	University of Delaware, Newark, Delaware, USA	University of Delaware, Newark, USA
	Negishi, Ei-ichi	Negishi, Heck, Suzuki “for palladium-catalyzed cross couplings in organic synthesis”	University of Pennsylvania, Philadelphia, USA	Syracuse University, Syracuse, New York, USA	Purdue University, West Lafayette, USA
	Suzuki, Akira	Suzuki, Heck, Negishi “for palladium-catalyzed cross couplings in organic synthesis”	Hokkaido University, Sapporo, Japan	Hokkaido University, Sapporo, Japan	Hokkaido University, Sapporo, Japan
2011	Shechtman, Daniel	Shechtman “for the discovery of quasicrystals”	Technion-Israel—Institute of Technology, Haifa, Israel	Technion-Israel—Institute of Technology, Haifa, Israel	Technion—Israel Institute of Technology, Haifa, Israel
2012	Lefkowitz, Robert Joseph	Lefkowitz, Kobilka “for studies of G-protein-coupled receptors”	Columbia University, New York, USA	Duke University, Durham, USA	Duke University Medical Center, Durham, USA
	Kobilka, Brian Kent	Kobilka, Lefkowitz “for studies of G-protein-coupled receptors”	Yale University, New Haven, USA	Duke University, Durham, USA	Stanford University, Stanford, USA
2013	Karplus, Martin	Karplus, Levitt, Warshel “for the development of multiscale models for complex chemical systems”	California Institute of Technology, (Caltech), Pasadena, USA	Harvard University, Cambridge, MA, USA	Université de Strasbourg, France and Harvard University, Cambridge, USA
	Levitt, Michael	Levitt, Warshall, Karplus “for the development of multiscale models for complex chemical systems”	Cambridge University, Medical Research Council (MRC), U.K	Medical Research Council (MRC), Cambridge University, U.K. and Weizmann Institute, Rohovot, Israel	Stanford University, School of Medicine, CA, USA
	Warshel, Arieh	Warshel, Levitt, Karplus “for the development of multiscale models for complex chemicalsystems”	Weizmann Instistute of Science (WIS), Rohovot, Israel	Medical Research Council (MRC), Cambridge University, U.K. and Weizmann Institute, Rehovot, Israel	University of Southern California (USC), Los Angeles, USA
2014	Betzig, Eric	Betzig, Hell, Moerner “for the development of super-resolved fluorescence microscopy”	Cornell University, Ithaka, NY, USA	AT&T Bell Laboratories, Murray Hill, NJ, USA	Janelia Research Campus, HHMI (Howard Hughes Medical Inst.), Ashburn, USA
	Hell, Stefan Walther	Hell, Betzig, Moerner “for the development of super-resolved fluorescence microscopy”	Ruprecht-Karls-Universität Heidelberg, Germany	University of Turku, Finnland	Max-Planck-Institute of biophysical chemistry (f. biophysikalische Chemie), Göttingen, Germany and German Cancer Research Center (DKFZ Deutsches Krebsforschungszentrum), Heidelberg, Germany
	Moerner, William Esco	Moerner, Hell, Betzig “for the development of super-resolved fluorescence microscopy”	Cornell University, Ithaka, NY, USA	University of California, San Diego, USA	Stanford University, CA, USA

**Table 6:** Detailed information on Nobel laureates in physiology/medicine

Year of award	Name	Justification	Ph.D. M.D. obtained	Did the prize-winning work(s)	Nobel Prize awarded
1994	Gilman, Alfred G.	Gilman, Rodbell: “for their discovery of G-proteins; and the role of these proteins in signal transduction in cells”	Case Western Reserve University, Cleveland, OH, USA M.D and Ph.D.	University of Virginia/University Texas, USA	University of Texas, Dallas, USA
	Rodbell, Martin	Rodbell, Gilman: “for their discovery of G-proteins; and the role of these proteins in signal transduction in cells”	University of Washington, Seattle, USA	National Institute of of Athritis, Metabolism and Digestive Diseases (NIAMD), Bethesta, USA	National Research Triangle Park (RTP), National Institute of Environmental Health Sciences (NIEHS), near Durham, NC, USA
1995	Lewis, Edward B.	Lewis, Nüsslein-Volhard, Wieschaus: “for their discoveries concerning the genetic control of early embryonic development”	California Institute of Technology (Caltech), Pasadena, USA	California Institute of Technology (Caltech), Pasadena, USA	California Institute of Technology (Caltech), Pasadena, USA
	Nüsslein-Volhard, Christiane	Nüsslein-Volhard, Lewis, Wieschaus: “for their discoveries concerning the genetic control of early embryonic development”	Eberhard-Karls-University Tübingen, Germany	European Molecular Biology Lab. (EMBL), Heidelberg, Germany	Max-Planck-Institute f. develomental biology (f. Entwicklungsbiologie), Tübingen, Germany
	Wieschaus, Eric F.	Wieschaus, Nüsslein-Volhard, Lewis: “for their discoveries concerning the genetic control of early embryonic development”	Yale University, CT, USA	European Molecular Biology Lab. (EMBL), Heidelberg, Germany	Princeton University, NJ, USA
1996	Doherty, Peter C.	Doherty, Zinkernagel: “for their discoveries concerning the specificity of the cell mediated immune defence”	University Edinburgh, U.K	Australian National University, Canberra, Australia	St. Jude Children’s Research Hospital, Memphis, Tennessee, USA
	Zinkernagel, Rolf M.	Zinkernagel, Doherty: “for their discoveries concerning the specificity of the cell mediated immune defence”	Australian National University, Canberra, Australia Ph.D., and M.D. University of Basel (Swiss)	Australian National University, Canberra, Australia	Institute of Exper. Immunology, Zürich, Swiss
1997	Prusiner Stanley B.	Prusiner “for his discovery of Prions—a new biological principle of infection”	University of California, San Francisco, USA	University of California, San Francisco, USA	University of California, San Francisco, USA
1998	Furchgott, Robert F.	Furchgott, Ignarro, Murad: “for their discoveries concerning nitric oxide as a signalling molecule in the cardio-vascular system”	Northwestern University Chicago, USA	State University of New York (SUNY) Brooklyn, New York, USA	State University of New York (SUNY) Brooklyn, New York, USA (retired) and University of Miami, FL, USA
	Ignarro, Louis J.	Ignarro, Furchgott, Murad: “for their discoveries concerning nitric oxide as a signalling molecule in the cardio-vascular system”	University Minnesota, Minneapolis-St. Paul, USA	University of California (UCLA), Los Angeles, USA	University of California (UCLA), Los Angeles, USA
	Murad, Ferid	Murad, Ignarro, Furchgott: “for their discoveries concerning nitric oxide as a signaling molecule in the cardio-vascular system”	Case Western Reserve University, Cleveland, OH, USA	University of Virginia, Charlottesville, USA	University of Texas, Houston, USA
1999	Blobel, Günter	Blobel: “for the discovery that proteins have intrinsic signals that govern their transport and localization in the cell”	Eberhard-Karls-University Tübingen, Germany (M.D.); University of Wisconsin, USA (Ph.D.)	Rockefeller University, Cell Biology Lab., New York, USA	Rockefeller University, New York, USA
2000	Carlsson, Arvid	Carlsson, Greengard, Kandel: “for their discoveries concerning signal transduction in the nervous system”	University of Lund, Sweden	University of Lund, Sweden	University of Göteborg, Sweden
	Greengard, Paul	Greengard, Carlsson, Kandel: “for their discoveries concerning signal trans-duction in the nervous system”	John Hopkins University, Baltimore, USA	Yale University, School of Medicine, USA	Rockefeller University, New York, USA
	Kandel, Eric R.	Kandel, Greengard, Carlsson: “for their discoveries concerning signal trans-duction in the nervous system”	New York University, School of Medicine, USA	New York University, School of Medicine, USA	Columbia University, New York, USA
2001	Hartwell, Leland H.	Hartwell, Hunt, Nurse: “for their discoveries of key regulators of the cell cycle”	Massachusetts Institute of Technology (MIT), Cambridge, USA	University of Washington, Seattle, USA	Fred Hutchinson Cancer Research Ctr. Seattle, USA
	Hunt, Timothy R.	Hunt, Hartwell, Nurse: “for their discoveries of key regulators of the cell cycle”	University of Cambridge, U.K	Marine Biology Lab, Woodshole, MA, USA	Imperial Cancer Research Fund, London, U.K
	Nurse, Paul M.	Nurse, Hunt, Hartwell: “for their discoveries of key regulators of the cell cycle”	University East Anglia (UEA), Norfolk, U.K	University of Edinburgh, U.K	Imperial Cancer Research Fund, London, U.K
2002	Brenner, Sydney	Brenner, Horvitz, Sulston: “for their discoveries concerning genetic regulation of organ development and ‘programmed cell ‘death’”	Oxford University, U.K	Medical Research Council (MRC), Cambridge, U.K	Molecular Sciences Institute, Berkeley, USA
	Horvitz, H. Robert	Horvitz, Brenner, Sulston: “for their discoveries concerning genetic regulation of organ development and ‘programmed cell ‘death’”	Harvard University, Cambridge, MA, USA	Medical Research Council (MRC) Cambridge, U.K	Massachusetts Institute of Technology (MIT), Cambridge, MA, USA
	Sulston, John E.	Sulston, Horvitz, Brenner: “for their discoveries concerning genetic regulation of organ development and ‘programmed cell ‘death’”	University of Cambridge, U.K	Medical Research Council (MRC) Cambridge, U.K	Sanger Institute, Cambridge, U.K
2003	Lauterbur, Paul C.	Lauterbur, Mansfield: “for their discoveries concerning magnetic resonance imaging”	University of Pittsburgh, PA, USA	State University of New York (SUNY), USA	University of Illinois, Urbana, USA
	Mansfield, Peter	Mansfield, Lauterbur: “for their discoveries concerning magnetic resonance imaging”	Queen Mary College, London, U.K	University of Nottingham, U.K	University of Nottingham, School of Physics and Astronomy, U.K
2004	Axel, Richard	Axel, Buck: “for their discoveries of odorant receptors and the organization of the olfactory system”	John Hopkins University, Baltimore, USA	Columbia University, New York, USA	Columbia University, New York, USA
	Buck, Linda B.	Buck, Axel: “for their discoveries of odorant receptors and the organization of the olfactory system”	University of Texas Southwestern Medical Center, Dallas, USA	Columbia University, New York, USA	Fred Hutchinson Cancer Research, Seattle, USA
2005	Marshall, Barry J.	Marshall, Warren: “for their discovery of the bacterium Helicobacter pylori and its role in gastritis and peptic ulcer disease”	University of Western Australia, Crawley, Australia	Royal Perth Hospital, Perth, Australia	NHMRC Helicobacter pylori Research Laboratory, QEII Medical Centre, Nedlands, Australia and University of Western Australia, Australia
	Warren, J. Robin	Warren, Marshall: “for their discovery of the bacterium Helicobacter pylori and its role in gastritis and peptic ulcer disease”	University of Adelaide, Australia	Royal Perth Hospital, Perth, Australia	Royal Perth Hospital, Perth, Australia
2006	Fire, Andrew Z.	Fire, Mello: “for their discovery of RNA interference—gene silencing by double-stranded RNA”	Massachusets Institute of Technology (MIT), Cambridge, MA, USA	Carnegie Institution f. Science, Baltimore, M.D., USA	Stanford University, CA, USA
	Mello, Craig C.	Mello, Fire: “for their discovery of RNA interference—gene silencing by double-stranded RNA”	Harvard University, Cambridge, MA, USA	University of Massachusetts, Worchester, USA	University of Massachusetts, Worchester, USA
2007	Capecchi, Mario R.	Capecchi, Evans, Smithies: “for their discoveries of principles for introducing specific gene modifications in mice by the use of embryonic stem cells”	Harvard University, Cambridge, MA, USA	University of Utah, Salt Lake City, USA	University of Utah, Salt Lake City, USA
	Evans, Martin J.	Evans, Capecchi, Smithies: “for their discoveries of principles for introducing specific gene modifications in mice by the use of embryonic stem cells”	University College London, U.K	University of Cambridge, U.K	Cardiff University, School of Biosciences, U.K
	Smithies, Oliver	Smithies, Evans, Capecchi: “for their discoveries of principles for introducing specific gene modifications in mice by the use of embryonic stem cells”	Oxford University, Balliol College, U.K	University of North Carolina, Chapel Hill, USA	University of North Carolina, Chapel Hill, USA
2008	Zur Hausen, H.	Zur Hausen: “for his discovery of human papilloma viruses causing cervical cancer”	Heinrich-Heine-Universität Düsseldorf (former Medical Academy), Germany	Albert-Ludwigs-University Freiburg, Germany	German cancer research center (DKFZ—Deutsches Krebsforschung Zentrum) Heidelberg, Germany
	Barré-Sinoussi, Françoise	Barré-Sinoussi, Montagnier: “for their discovery of human immuno-deficiency virus”	Institute Pasteur, Paris, France and University of Sciences, Paris, France	Institute Pasteur, Paris, France	Institute Pasteur, Regulation of Retroviral Infections Unit, Virology depart., Paris, France
	Montagnier, Luc	Montagnier, Barré-Sinoussi: “for their discovery of human immuno-deficiency virus”	University Sorbonne, Paris, France	Institute Pasteur, Paris, France	World Foundation AIDS Research and Prevention Paris, France
2009	Blackburn, Elisabeth H.	Blackburn, Greider, Szostak: “for the discovery of how chromosomes are protected by telomeres and the enzyme telomerase”	University of Cambridge, U.K	University of California, Berkeley, USA	University of California, Berkeley, USA
	Greider, Carol W.	Greider, Blackburn, Szostak: “for the discovery of how chromosomes are protected by telomeres and the enzyme telomerase”	University of California, Berkeley, USA	University of California, Berkeley, USA	John Hopkins, School of Medicine, Baltimore, USA
	Szostak, Jack W.	Szostak, Greider, Blackburn: “for the discovery of how chromosomes are protected by telomeres and the enzyme telomerase”	Cornell University, New York, USA	Harvard University, School of Medicine, Cambridge, MA, USA	Harvard University, Cambridge, MA, USA
2010	Edwards, Robert G.	Edwards: “for the development of in vitro fertilization”	University of Edinburgh, U.K	University of Cambridge, U.K	University of Cambridge, U.K
2011	Beutler, Bruce A.	Beutler, Hoffmann: “for their discoveries concerning the activation of innate immunity”	University Chicago, IL, USA	Rockefeller University, New York, USA	University of Texas, Dallas, USA and The Scripps Research Institute, La Jolla, CA, USA
	Hoffmann, Jules A.	Hoffmann, Beutler: “for their discoveries concerning the activation of innate immunity”	University Strasbourg, France	French National Center for Scientific Research (CNRS) Strasbourg, France	French National Center for Scientific Research (CNRS) Strasbourg, France
	Steinman, Ralph M.	Steinman: “for his discovery of the dendritic cell and its role in adaptive immunity”	Harvard University, Cambridge, USA	Rockefeller University, New York, USA	Rockefeller University, New York, USA
2012	Gurdon, John B.	Gurdon, Yamanaka: “for the discovery that mature cells can be reprogrammed to become pluripotent”	Oxford University, U.K	Oxford University, U.K	Cambridge University, U.K
	Yamanaka, Shinya	Yamanaka, Gurdon: “for the discovery that mature cells can be reprogrammed to become pluripotent”	Osaka City University, Osaka, Japan Ph.D. and Kobe University, Japan M.D.	Kyoto University, Kyoto, Japan and CREST, Japan and Science and Technology Agency, Kawaguchi, Japan	Kyoto University, Kyoto, Japan
2013	Rothman, James E.	Rothman, Schekman, Südhof: “for their discoveries of machinery regulating vesicle traffic, a major transport system in our cells”	Harvard University, Cambridge, MA, USA	Memorial Sloan Kettering Cancer Center, New York, USA	Yale University, New Haven, USA
	Schekman, Randy W.	Schekman, Rothman, Südhof: “for their discoveries of machinery regulating vesicle traffic, a major transport system in our cells”	Stanford University, CA, USA	University of California, Berkeley, USA	University of California, Berkeley, USA
	Südhof, Thomas C.	Südhof, Schekman, Rothman: “for their discoveries of machinery regulating vesicle traffic, a major transport system in our cells”	Max-Planck-Institute f. biophysical chemistry (f. biophysikalische Chemie), Göttingen, Germany and Georg-August-Universität, Göttingen, Germany	University of Texas, Dallas, USA	Stanford University, CA, USA
2014	O’Kneefe, John M.	O’Kneefe: “for their discoveries of cells that constitute a positioning system in the brain”	McGill University, Montreal, Canada	University College London (UCL), London, U.K	University College London (UCL), U.K
	Moser, May-Britt	Moser M, Moser E: “for their discoveries of cells that constitute a positioning system in the brain”	University of Oslo, Norway	Norwegian University of Science and Technology (NTNU), Trondheim, Norway	Norwegian University of Science and Technology (NTNU), Trondheim, Norway
	Moser, Edvard I.	Moser E, Moser M: “for their discoveries of cells that constitute a positioning system in the brain”	University of Oslo, Norway	Norwegian University of Science and Technology (NTNU), Trondheim, Norway	Norwegian University of Science and Technology (NTNU), Trondheim, Norway

**Table 7:** Detailed information on Nobel laureates in physics

Year of award	Name	Justification	Ph.D./M.D. obtained	Did the prize-winning work(s)	Nobel Prize awarded
1994	Brockhouse, Bertram N.	Brockhouse, Shull: “for pioneering contributions to the development of neutron scattering techniques for studies of condensed matter”. Brockhouse “for the development of neutron spectroscopy”	University of Toronto, Canada	National Reactor Universal (NRU), Chalk River Laboratories (CRL), facility from Atomic Energy of Canada Limeted (AECL), Chalk River, Canada	McMaster University, Hamilton, Ontario, Canada
	Shull, Clifford G.	Brockhouse, Shull: “for pioneering contributions to the development of neutron scattering techniques for studies of condensed matter”. Shull: “for the development of the neutron diffraction technique”	New York University, USA	Oak Ridge National Laboratories, Oak Ridge, near Knoxville, Tennessee, USA	Massachusetts Institute of Technology (MIT), Cambridge, MA, USA
1995	Perl, Martin L.	Perl, Reines: “for pioneering experimental contributions to lepton physics”. Perl “for the discovery of the tau lepton”	Columbia University, New York, USA	Stanford University, CA, USA	Stanford University, CA, USA
	Reines, Frederick	Perl, Reines: “for pioneering experimental contributions to lepton physics”. Reines “for the detection of the neutrino”	New York University, USA	University of California, Los Alamos, Scientific Laboratory, USA	University of California, Irvine (UCI), USA
1996	Lee, David M.	Lee, Osheroff, Richardson: “for their discovery of superfluidity in helium-3”	Yale University, New Haven, USA	Cornell University, Ithaca, USA	Cornell University, Ithaca, USA
	Osheroff, Douglas D.	Osheroff, Lee, Richardson: “for their discovery of superfluidity in helium-3”	Cornell University, Ithaca, NY, USA	Cornell University, Ithaca, NY, USA	Stanford University, CA, USA
	Richardson, Robert C.	Richardson, Lee, Osheroff: “for their discovery of superfluidity in helium-3”	Duke University, Durham, USA	Cornell University, Ithaca, NY, USA	Cornell University, Ithaca, NY, USA
1997	Chu, Steven	Chu, Cohen-Tannoudji, Phillips: “for development of methods to cool and trap atoms with laser light”	University of California, Berkeley, USA	Bell Laboratories (AT&T), room Alcatel -Lucent, Head office Murray Hill, New Jersey, USA	Stanford University, CA, USA
	Cohen-Tannoudji, Claude	Cohen-Tannoudji, Chu, Phillips: “for development of methods to cool and trap atoms with laser light”	École Normale Superiéure (ENS), Paris, France	Université de Paris 07, École Normale Supérieure, Paris, France.	Collège de France, Paris, France and École Normale Supérieure, Paris, France
	Phillips, William D.	Phillips, Chu, Cohen-Tannoudji: “for development of methods to cool and trap atoms with laser light”	Massachusetts Institute of Technology, (MIT), Cambridge, USA	National Bureau of Standards (now the National Institute of Standards and Technology, NIST), Gaithersburg, MD, USA	National Institute of Standards and Technology (NIST), Gaithersburg, MD, USA
1998	Laughlin, Robert B.	Laughlin, Störmer, Tsui: “for their discovery of a new form of quantum fluid with fractionally charged excitations”	Massachusetts Institute of Technology, (MIT), Cambridge, USA	University of California, The Lawrence Livermore National Laboratory, Livermore, USA	Stanford University, Palo Alto, CA, USA
	Störmer, Horst L.	Störmer, Laughlin, Tsui: “for their discovery of a new form of quantum fluid with fractionally charged excitations”	Universität Stuttgart, Federal Republique of Germany, BRD	Bell Laboratories (now: AT&T), Murray Hill, NJ, USA	Columbia University, New York, USA
	Tsui, Daniel C.	Tsui, Störmer, Laughlin: “for their discovery of a new form of quantum fluid with fractionally charged excitations”	University of Chicago, IL, USA	Bell Laboratories (now: AT&T), Murray Hill, NJ, USA	Princeton University, NJ, USA
1999	_’T Hooft, Gerardus	_T’Hooft, Veltman: “for elucidating the quantum structure of electroweak interactions in physics”	Utrecht University, The Netherlands	Utrecht University, The Netherlands	Utrecht University, The Netherlands
	Veltman, Martinus J. G.	Veltman, Hooft: “for elucidating the quantum structure of electroweak interactions in physics”	Utrecht University, The Netherlands	Utrecht University, The Netherlands	University of Michigan, Ann Arbor, USA (retired)
2000	Alferov, Zhores (Schores) Ivanovich	Zhores, Kroemer, Kilby: “for basic work on information and communication technology”. Zhores, Kroemer: “for developing semiconductor heterostructures used in high-speed- and opto-electronics”	Electrotechnical Institute, (former depart. of Electronics of V. I. Ulyanov (Lenin), St. Petersburg, Russia	A.F. Ioffe Physico-Technical Institute, St. Petersburg, Russia	A.F. Ioffe Physico-Technical Institute, St. Petersburg, Russia
	Kroemer, Herbert	Kroemer, Zhores, Kilby: “for basic work on information and communication technology”. Kroemer, Zhores: “for developing semiconductor heterostructures used in high-speed- and opto-electronics”	Georg-August-University Göttingen, Germany	Varian Associates, Paolo Alto, CA, USA	University of California, Santa Barbara, USA
	Kilby, Jack S.	Kilby, Kroemer, Zhores: “for basic work on information and communication technology”. Kilby: “for his part in the invention of the integrated circuit”	No Ph.D. (Master`s degree University of Illinois, Illinois, USA)	Texas Instruments Incooperated (Bell liscensee), Dallas, USA	Texas Instruments Incooperated, Dallas, USA
2001	Cornell, Eric A.	Cornell, Ketterle, Wieman: “for the achievement of Bose-Einstein condensation in dilute gases of alkali atoms, and for early fundamental studies of the properties of the condensates”	Massachusetts Institute of Technology (MIT), Cambridge, USA	University of Colorado, Joint Institute for Laboratory Astrophysics (JILA), Boulder, CO, USA	University of Colorado, Joint Institute of Laboratory of Astrophysics (JILA), Boulder, CO, USA
	Ketterle, Wolfgang	Ketterle, Cornell, Wieman: “for the achievement of Bose-Einstein condensation in dilute gases of alkali atoms, and for early fundamental studies of the properties of the condensates”	Technische Universität (TU) Munich, Germany and Max-Planck-Institute of quantumoptics (f. Quantenoptik), Garching, Germany	Massachusetts Institute of Technology (MIT), Cambridge, MA, USA	Massachusetts Institute of Technology (MIT), Cambridge, MA, USA
	Wieman, Carl E.	Wieman, Ketterle, Cornell: “for the achievement of Bose–Einstein condensation in dilute gases of alkali atoms, and for early fundamental studies of the properties of the condensates”	Stanford University, CA, USA	University of Colorado, Joint Institute for Laboratory Astrophysics (JILA), Boulder, CO, USA	University of Colorado, Joint Institute for Laboratory Astrophysics (JILA), Boulder, CO, USA
2002	Davis, Raymond Jr.	Koshiba, Davis: “for pioneering contributions to astrophysics, in particular for the detection of cosmic neutrinos”	Yale University, New Haven, CT, USA	Brookhaven National Laboratory, Upton, New York, USA	University of Pennsylvania, Philadelphia, USA
	Koshiba, Masatoshi	Koshiba, Davis: “for pioneering contributions to astrophysics, in particular for the detection of cosmic neutrinos”	University of Rochester, New York, USA	University of Tokyo, Japan	University of Tokyo, Japan
	Giacconi, Riccardo	Giacconi: “for pioneering contributions to astrophysics, which have led to the discovery of cosmic X-ray sources”	Università degli studi di Milano, Milan, Italy	American Science and Enginerring, Inc., Cambridge, MA, USA	Assoc. Universities Inc., Washington DC., USA
2003	Abrikosov, Alexei A.	Abrikosov, Ginzburg, Leggett: “for pioneering contributions to the theory of superconductors and superfluids”	Institute for Physical Problems (now the P.L. Kapitsa Institute), Moscow, Russia	P.L. Kapitsa Institute, Moscow, Russia	Argonne National Laboratory, Argonne, USA
	Ginzburg, Vitaly L.	Ginzburg, Abrikosov, Leggett: “for pioneering contributions to the theory of superconductors and superfluids”	Moscow State University, Russia	P.N. Lebedev Physical Institute of the U.S.S.R. Academy of Sciences, Moscow, Russia	P.N. Lebedev Physical Institute of the U.S.S.R. (now Russian) Academy of Sciences, Moscow, Russia
	Leggett, Anthony J.	Leggett, Ginzburg, Abrikosov: “for pioneering contributions to the theory of superconductors and superfluids”	University of Oxford, U.K	University of Sussex, Brighton, U.K	University of Illinois, Urbana, USA
2004	Gross, David J.	Gross, Politzer, Wilczek: “for the discovery of asymptotic freedom in the theory of the strong interaction”	University of California, Berkeley, USA	Fermi National Accelarator Laboratory (Fermilab), Batavia, IL, USA and Princeton University, Joseph Henry Laboratory, NC, USA	University of California, Kavli Institute for Theoretical Physics, Santa Barbara, USA
	Politzer, Hugh D.	Politzer, Gross, Wilczek: “for the discovery of asymptotic freedom in the theory of the strong interaction”	Harvard University, Cambridge, MA, USA	Harvard University, Cambridge, Massachusetts, USA	California Institute of Technology (Caltech), Pasadena, USA
	Wilczek, Frank	Wilczek, Gross, Politzer: “for the discovery of asymptotic freedom in the theory of the strong interaction”	Princeton University, NC, USA	Princeton University, NC, USA	Massachusetts Institute of Technology (MIT), Cambridge, MA, USA
2005	Glauber, Roy J.	Glauber: “for his contribution to the quantum theory of optical coherence”	Harvard University, Cambridge, MA, USA	Harvard University, Cambridge, MA, USA	Harvard University, Cambridge, MA, USA
	Hall, John L.	Hall, Hänsch: “for their contributions to the development of laser-based precision spectroscopy, including the optical frequency comb technique”	Carnegie Institute of Technology, Pittsburgh, USA	University of Colorado, Joint Institute for Laboratory Astrophysics (JILA), Boulder, CO, USA	University of Colorado, Joint Institute for Laboratory Astrophysics (JILA), Boulder, CO, USA and The National Institute of Standards and Technology, NIST, Boulder, CO, USA
	Hänsch, Theodor W.	Hänsch, Hall: “for their contributions to the development of laser-based precision spectroscopy, including the optical frequency comb technique”	Ruprecht-Karls-Universität Heidelberg, Germany	Max-Planck-Institute of quantum optics (f. Quantumoptik), Garching, Germany	Max-Planck-Institute of quantum optics (f. Quantenoptik), Garching, Germany and Ludwig-Maximlians-University, Munich, Germany
2006	Mather, John C.	Mather, Smoot: “for their discovery of the blackbody form and anisotropy of the cosmic microwave background radiation”	University of California, Berkeley, USA	NASA Goddard Institute for Space Flight Center, Greenbelt, USA	NASA Goddard Space Flight Center, Greenbelt, USA
	Smoot, George F.	Smoot, Mather: “for their discovery of the blackbody form and anisotropy of the cosmic microwave background radiation”	Massachusetts Institute of Technology (MIT), Cambridge, USA	University of California, Berkeley, USA	University of California, Berkeley, USA
2007	Fert, Albert	Fert, Grünberg: “for the discovery of Giant Magnetoresistance”	Université Paris-Sud, Orsay, France	Université Paris-Sud, Orsay, France	Université Paris-Sud, Orsay, France, Unité Mixte de Physique CNRS/THALES, Orsay, France
	Grünberg, Peter A.	Grünberg, Fert: “for the discovery of Giant Magnetoresistance”	Technische Universität (TU), Darmstadt, Germany	Helmholtz IFF-Forschungszentrum Jülich, Institut f. Festkörperforschung (now: Peter Grünberg Institute), Germany	Helmholtz IFF-Forschungszentrum Jülich, Germany
2008	Nambu, Yoichiro	Nambu: “for the discovery of the mechanism of spontaneous broken symmetry in subatomic physics”	University of Tokyo, Japan	University of Chicago, Enrico Fermi Institute, IL, USA	University of Chicago, Enrico Fermi Institute, IL, USA
	Kobayashi, Makoto	Kobayashi, Maskawa: “for the discovery of the origin of the broken symmetry which predicts the existence of at least three families of quarks in nature”	Nagoya University, Japan	Kyoto University, Japan	High Energy Accelerator Research Organization (KEK), Tsukuba, Japan
	Maskawa, Toshihide	Maskawa, Kobayashi: “for the discovery of the origin of the broken symmetry which predicts the existence of at least three families of quarks in nature”	Nagoya University, Japan	Kyoto University, Japan	Kyoto Sangyo University, Japan and Yukawa Institute for Theoretical Physics (YITP), Kyoto University, Japan
2009	Kao, Charles Kuen	Koa: “for groundbreaking achievements concerning the transmission of light in fibers for optical communication”	University of London, College of London, U.K	Standard Telecommunication Laboratories (STL), Harlow, Essex, U.K	Standard Telecommunication Laboratories (STL), Harlow, Essex, U.K. and Chinese University of Hong Kong, Hong Kong, China
	Boyle, Willard S.	Boyle, Smith: “for the invention of an imaging semiconductor circuit-the CCD sensor”	McGill University, Montreal, Quebec, Canada	Bell Laboratories (now: AT&T), Murray Hill, NJ, USA	Bell Laboratories (now: AT&T), Murray Hill, NJ, USA
	Smith, George E.	Smith, Boyle: “for the invention of an imaging semiconductor circuit-the CCD sensor”	University of Chicago, USA	Bell Laboratories (now: AT&T), Murray Hill, NJ, USA	Bell Laboratories (now: AT&T), Murray Hill, NJ, USA
2010	Geim, Andre K	Geim, Novoselov: “for groundbreaking experiments regarding the two-dimensional material graphene”	Institute of Solid State Physics, Chernogolovka, near Moscow, Russia	University of Manchester, U.K	University of Manchester, U.K
	Novoselov, Konstantin S.	Novoselov, Geim: “for groundbreaking experiments regarding the two-dimensional material graphene”	Radboud University Nijmegen, The Netherlands	University of Manchester, U.K	University of Manchester, U.K
2011	Perlmutter, Saul	Perlmutter: “for the discovery of the accelerating expansion of the Universe through observations of distant supernovae”	University of California, Berkley, USA	University of California, Lawrence Berkeley National Laboratory, Berkeley, USA	University of California, Lawrence Berkeley National Laboratory, Berkeley, USA
	Schmidt, Brian P.	Schmidt, Riess: “for the discovery of the accelerating expansion of the Universe through observations of distant supernovae”	Harvard University, Cambridge, MA, USA	Australian National University (MSSSO Moint Strongly and Siding Spring Observations), Weston Creek, Australia	Australian National University (MSSSO Moint Strongly and Siding Spring Observations), Weston Creek, Australia
	Riess, Adam Guy	Riess, Schmidt: “for the discovery of the accelerating expansion of the Universe through observations of distant supernovae”	Harvard University, Cambridge, MA, USA	University of California, Berkeley, USA	John Hopkins University, Space Telescope Science Institute, Baltimore, MD, USA
2012	Haroche, Serge	Haroche, Wineland: “for ground-breaking experimental methods that enable measuring and manipulation of individual quantum systems”	Université Paris VI (now Université Pierre et Marie Curie), Paris, France	École Normale Supérieure, Lab. Kastler Brossel, Paris, France	Collège de France, Paris, France and École Normale Supérieure, Paris, France
	Wineland, David J.	Wineland, Haroche: “for ground-breaking experimental methods that enable measuring and manipulation of individual quantum systems”	Harvard University, Cambridge, MA, USA	University of Colorado, Boulder, CO and NIST National Institute of Standards and Technology, Boulder, CO, USA	University of Colorado, Boulder, CO, USA and NIST National Institute of Standards and Technology, Boulder, CO, USA
2013	Englert, François	Englert, Higgs: “for the theoretical discovery of a mechanism that contributes to our understanding of the origin of mass of subatomic particles, and which recently was confirmed through the discovery of the predicted fundamental particle, by the ATLAS and CMS experiments at CERN’s Large Hadron Collider”	Université Libre de Bruxelles (ULB), Belgium	Université Libre de Bruxelles (ULB), Belgium	Université Libre de Bruxelles (ULB), Belgium
	Higgs, Peter W.	Higgs, Englert: “for the theoretical discovery of a mechanism that contributes to our understanding of the origin of mass of subatomic particles, and which recently was confirmed through the discovery of the predicted fundamental particle, by the ATLAS and CMS experiments at CERN’s Large Hadron Collider”	University of London, King’s College London, London, U.K	University of Edinburgh, Scotland, U.K	University of Edinburgh, Scotland, U.K
2014	Akasaki, Isamu	Akasaki, Amano, Nakamura: “for the invention of efficient blue light-emitting diodes which has enabled bright and energy-saving white light sources”	Nagoya University, Japan	Nagoya University, School of Engineering, Japan	Meijo University, Nagoya, Japan and University Nagoya, Japan
	Amano, Hiroshi	Amano, Akasaki, Nakamura: “for the invention of efficient blue light-emitting diodes which has enabled bright and energy-saving white light sources”	Nagoya University, Japan	Nagoya University, School of Engineering, Japan	Nagoya University, Japan
	Nakamura, Shuji	Nakamura, Amano, Akasaki: “for the invention of efficient blue light-emitting diodes which has enabled bright and energy-saving white light sources”	University of Tokushima, Japan	University of Tokushima, Japan	University of California, Santa Barbara, USA
